# Close Encounters of Lymphoid Cells and Bacteria

**DOI:** 10.3389/fimmu.2016.00405

**Published:** 2016-10-07

**Authors:** Aranzazu Cruz-Adalia, Esteban Veiga

**Affiliations:** ^1^Department of Molecular and Cellular Biology, Centro Nacional de Biotecnología, Consejo Superior de Investigaciones, Científicas (CNB-CSIC), Madrid, Spain

**Keywords:** innate-like lymphocytes, conventional T cells, unconventional T cells, gamma delta T cells, B cells, bacteria–lymphocyte interactions

## Abstract

During infections, the first reaction of the host against microbial pathogens is carried out by innate immune cells, which recognize conserved structures on pathogens, called pathogen-associated molecular patterns. Afterward, some of these innate cells can phagocytose and destroy the pathogens, secreting cytokines that would modulate the immune response to the challenge. This rapid response is normally followed by the adaptive immunity, more specific and essential for a complete pathogen clearance in many cases. Some innate immune cells, usually named antigen-presenting cells, such as macrophages or dendritic cells, are able to process internalized invaders and present their antigens to lymphocytes, triggering the adaptive immune response. Nevertheless, the traditional boundary of separated roles between innate and adaptive immunity has been blurred by several studies, showing that very specialized populations of lymphocytes (cells of the adaptive immunity) behave similarly to cells of the innate immunity. These “innate-like” lymphocytes include γδ T cells, invariant NKT cells, B-1 cells, mucosal-associated invariant T cells, marginal zone B cells, and innate response activator cells, and together with the newly described innate lymphoid cells are able to rapidly respond to bacterial infections. Strikingly, our recent data suggest that conventional CD4^+^ T cells, the paradigm of cells of the adaptive immunity, also present innate-like behavior, capturing bacteria in a process called transinfection. Transinfected CD4^+^ T cells digest internalized bacteria like professional phagocytes and secrete large amounts of proinflammatory cytokines, protecting for further bacterial challenges. In the present review, we will focus on the data showing such innate-like behavior of lymphocytes following bacteria encounter.

## Introduction

Classically, the immune system is classified into innate and adaptive immunity. During pathogen challenges, the first host defense involves the innate immune system that provides an immediate response. Innate immune systems are widely spread in nature and can be found in all plants and animals (1). Cells of the innate immune system include mast cells, eosinophils, basophils, natural killers (NKs), and phagocytes [macrophages, neutrophils, and dendritic cells (DCs)]. These cells recognize conserved structures, shared by different pathogens, called pathogen-associated molecular patterns (PAMPs) by their pattern-recognition receptors (PRRs). Afterward, they eliminate pathogens, either by combating through contact or by engulfing them. Some of these phagocytes are also antigen-presenting cells (APCs), such as DCs, and after engulfment pathogens, they are able to process and present the invader antigens to activate the cells of the adaptive immunity, the lymphocytes. Albeit both innate and adaptive immunity can distinguish between self and non-self molecules, adaptive immunity is defined by its capacity to specifically recognize a large amount of different antigens, defined by any non-self substance that can be recognized by the immune system. Theoretically, more than 10^13^ different antigens could be recognized by the adaptive immunity (2). This highly specific adaptive response takes more time to occur and generates memory, i.e., a second exposition to the same antigen results in faster and more potent response.

B and T cells are the major types of lymphocytes within the adaptive response and need to be activated by professional APCs during the antigen presentation. Regarding T cell activation, processed antigens are presented into the major histocompatibility complex (MHC) molecules on the membrane of APCs. There are two main subtypes of T cells: helper T cells (CD4^+^) and cytotoxic T cells (CD8^+^). Activation of CD4^+^ T cells occurs by the recognition of antigens coupled to class II MHC molecules (MHC-II) by T cell receptor (TCR). Typically, MHC-II molecules expose antigens degraded into lysosomal compartments (i.e., “foreign” antigens). On the other hand, CD8^+^ T cells are activated by the detection of antigens coupled to class I MHC (MHC-I). MHC-I molecules present antigens from the cytoplasm (i.e., self-antigens, or viral antigens), but DCs, are also able to present foreign antigens, degraded the lysosome, in their MHC-I by a process called cross-presentation, which is of the major relevance in antibacterial and antitumor fight. B cells express B cell receptors (BCRs) that recognize soluble molecules from pathogens with no need for antigen processing. It has been shown, however, that B cell activation requires presentation by professional APCs *in vivo* (3). This presentation does not require MHC molecules.

Antigen presentation by APCs triggers activation and differentiation of naïve lymphocytes to effector cells. B cells suffer immunoglobulin isotype switching and somatic hypermutation, which increase the affinity of the antibodies, and T cells develop distinct effector functions (for example, the secretion of a different array of cytokines or cytolytic activity). This textbook view of the innate and adaptive immunity role separation is being blurred by the discovery of lymphoid cells behaving in an innate-like manner (4, 5). Similarly, there exists an increasing body of evidences showing that cells of the innate immunity present adaptive-like behavior developing memory-like characteristics, termed “trained immunity.” Trained monocytes respond more efficiently to a second exposition of the same (and different) challenges (6).

In this review, we will focus on the innate-like role of lymphoid cells. These innate-like lymphocytes include specialized populations of lymphocytes, i.e., unconventional (γδ) T cells, invariant NKT cells (iNKT), mucosal-associated invariant T (MAIT) cells, B-1 cells, marginal zone (MZ) B cells, innate response activator (IRA) B cells, and the innate lymphoid cells (ILCs) (4). Surprisingly, we have recently shown that conventional αβ CD4^+^ T cells, paradigm of adaptive immune cells, are able to capture bacteria from DCs in a process called transinfection and contribute to the early immune response (7). Here, we discuss in some detail the innate-like functions performed by different types of lymphocytes during bacteria encounter.

### γδ T Cells

These T cells, expressing the unconventional γδ TCR, were discovered from the accidental identification of the TCRγ chain (8). γδ TCRs and αβ TCRs have qualitatively distinct modes of antigen recognition; γδ TCRs are not restricted to the recognition of peptides bound to MHC molecules (9). Unlike conventional αβ T cells, cytokine stimulation, or bacterial contact, is sufficient for activation γδ T cells, making these cells rapid and potent mediators of inflammation.

They are much less abundant than classical αβ T cells (1–4%) in thymus and lymphoid organs of adult mice, but they are in highest abundance in mucosal sites, being ~20–40% of the intestinal intraepithelial T cells, ~10–20% of total T cells in the reproductive tracks, and ~50–70% of skin dermal T cells (10).

In humans, the population of peripheral blood γδ T cells is increased in response to infections (11). Initial characterization of human γδ T cells suggested that antigens recognized by γδ T cells were small, non-peptide compounds that contained critical phosphate residues (12). The mainstream γδ T cells in human peripheral blood express the TCR Vγ9Vδ2, and they can recognize (E)-4-hydroxy-3-methyl-but-2enyl pyrophosphate (HMBPP), which are usually referred as phosphoantigens, derived from various bacteria (13). Moreover, human Vγ2Vδ2^+^ T cells can expand 2- to 10-fold during infections and recognize primary alkylamines derived from microbes, releasing interleukine-2 (IL-2) (14). Lysates or culture supernatants from many bacteria (including mycobacteria, other Gram-negative and Gram-positive cocci, protozoal parasites, and even plants extracts) stimulate Vγ2Vδ2^+^ T cells (15). Thus, human peripheral blood γδ T cells can respond to specific antigens from bacteria [e.g., *Mycobacterium tuberculosis* (16) and *Listeria monocytogenes* (17)]. Non-peptidic mycobacterial ligands in human Vγ9Vδ2^+^ T cells induce massive tumor necrosis factor (TNF) production (18). Moreover, Vγ2Vδ2^+^ T cells respond to non-peptide bacterial antigens predominantly producing Th1 cytokines such as interferon-γ (IFN-γ), although few of them (<5%) also produce IL-4 (15). It has been reported that *Helicobacter pylori* can directly interact with human peripheral γδ T cells *in vitro*, upregulating the activation molecule CD69, TNF-α, IFN-γ, and chemokines, such as MIP-1β and RANTES, favoring an inflammatory environment (19).

In mice, γδ T cells expand dramatically after challenge with *Mycobacteria, Listeria*, and *Salmonella* spp. (20–22), rapidly producing cytokines. They are able to produce IFN-γ after *L. monocytogenes* infection and IL-4 in response to *Nippostrongylus brasiliensis* (23). Moreover, it has also been reported that γδ T cells are the major IL-17 producers during infections (24, 25).

#### Toll-Like Receptors in γδ T Cells

Recognition of bacterial PAMPs by innate immune cells is driven mainly by TLRs, a type of PRRs that recognize bacterial patterns including peptidoglycan and bacterial lipopeptides (TLR1, 2, and 6), lipopolysaccharide (LPS) (TLR4), or flagellin (TLR5), others recognize nucleic acid including double-stranded viral RNA (TLR3), single-stranded RNA (TLR7 and 8), or unmethylated bacterial DNA (TLR9). Both human and mouse γδ T cells express functional TLRs. There are several studies showing TLR2 expression on γδ T cells in mice (26, 27) and humans (28), supporting a role in early responses to bacterial infections.

In mice, it has been shown that CCR6^+^IL-17-producing γδ T cells, but not other γδ T cells, express TLR1 and TLR2, as well as dectin-1, and could directly interact with certain pathogens. Toll-like receptor (TLR) stimulation in synergy with IL-23 results in cell expansion, IL-17 production, and further recruitment of neutrophils *in vivo* (29). In addition, the expression of TLR1, TLR2, TLR-6, TLR-9, and even TLR-4 by mice γδ T cells has been confirmed (30). Furthermore, it has been shown that IL-23 stimulation of splenic γδ, but not αβ, T cells leads to enhanced TLR1, -2, and -4 mRNA expression. TLR2 agonist Pam3CSK4, but not IL-23, stimulates splenic γδ T cell expansion *in vitro* (30). However, TLR agonist Pam3CSK4 and other pathogen products alone do not stimulate dermal γδ T cell proliferation, which require IL-23 (31). Additionally, TLR agonists Pam3CSK4 (TLR2), Gardiquimod (TLR7), and CpG (TLR9), but not LPS (TLR4) or dectin-1 ligand curdlan, stimulate dermal γδ T cells to produce IL-17, which is enhanced in the presence of IL-23 (31). It has also been reported that TLR4 is involved in the production of IL-17 and IFN-γ by γδ T cells during experimental autoimmune encephalomyelitis (EAE) induction (32).

In humans, the two major γδ T cell subsets, Vδ1 and Vδ2, express TLR1, TLR2, and TLR3 (33). Indeed, it has been demonstrated that human γδ T cells isolated from blood express high levels of TLR2, and its engagement promotes the release of IFNγ (28). Furthermore, the expression of TLR3 on human γδ cells has been verified by flow cytometry and confocal microscopy (34). Human γδ T cells secrete IFN-γ and upregulate CD69 after stimulation *via* TCR in the presence of poly (I:C), a TLR-3 agonist, without APC engagement (34).

**Figure 1:**
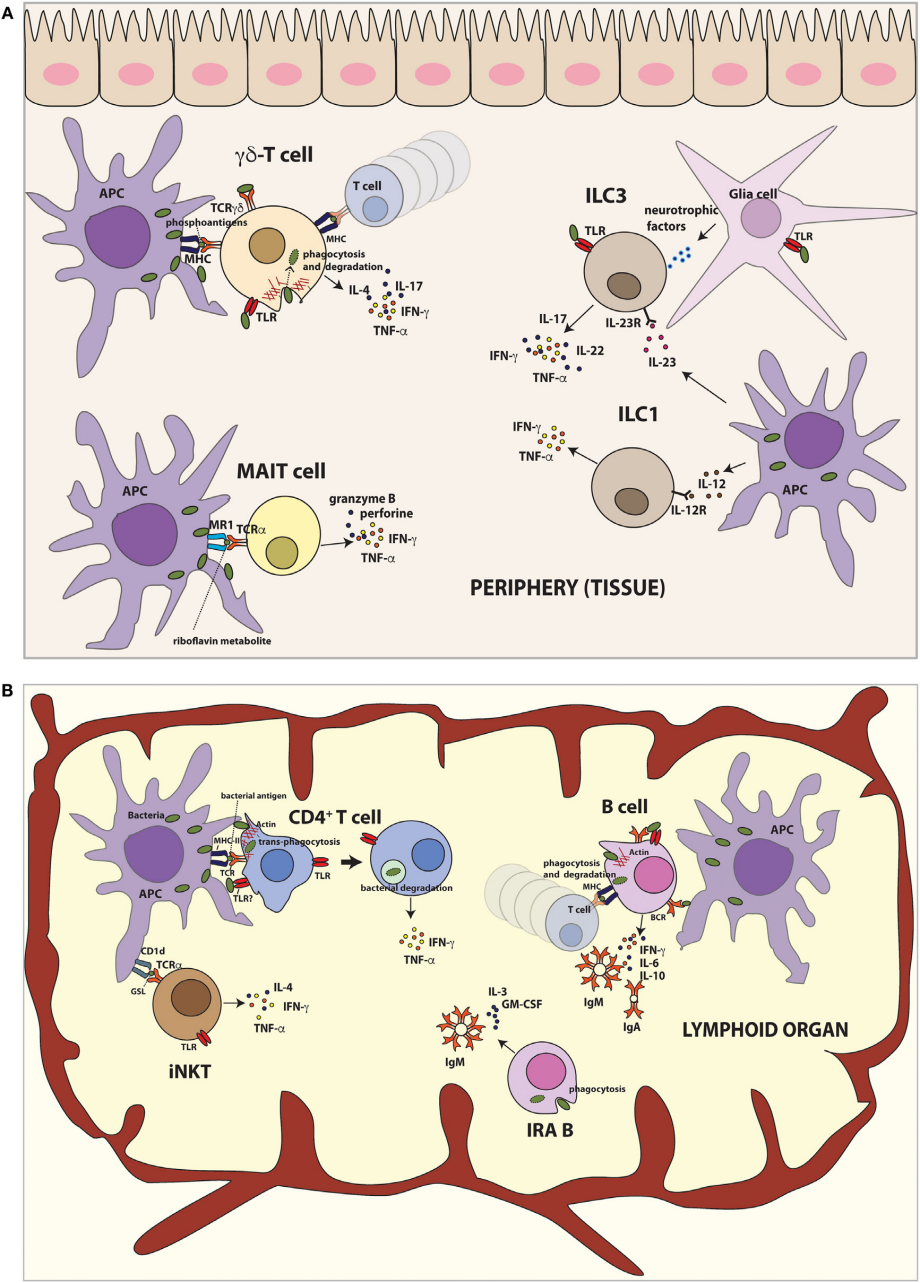
**Innate-like functions by different lymphoid cells after bacteria encounter**. **(A)** Lymphoid populations in peripheral tissues. γδ T cells recognize non-peptide phosphoantigens derived from bacteria and induce production of TNF-α, IFN-γ, IL-4, and IL-17. Moreover, human γδ T cells are able to phagocytose bacteria and present bacterial antigens on MHC class II to activate T cells *in vitro*. TLR stimulation of γδ T cells also results in IL-17 and IFN-γ production. MAIT cells recognize microbial riboflavin metabolites presented by MR1 and produce TNF-α and IFN-γ. Furthermore, human MAIT cells destroy infected cells by secretion of cytotoxic granzyme and perforin. ILC1 cells promote defense against intracellular bacteria by TNF-α and IFN-γ. ILC3 cells respond to extracellular bacteria, inducing mainly IL-17 and IL-22. TLR stimulation of ILC3 cells also results in secretion of proinflammatory cytokines such as TNF-α and IFN-γ production. Mouse ILC3 cells can form a functional unit with glial cells in the epithelial gut sensing the environment in TLR–MyD88-dependent manner and control the immune response *via* IL-22 secretion. **(B)** Lymphoid populations in lymphoid organs. iNKT cells recognize bacterial glycosphingolipid (GSL) presented by CD1d and produce IFN-γ. Additionally, TLR engagement of iNKT cells leads to IFN-γ, IL-4, and TNF-α production. B cells capture soluble antigens or antigens exposed by APCs. On the other hand, they can phagocytose bacteria and present bacterial antigens to T lymphocytes. After bacteria encounter, B cells secrete large amount of IgM and/or IgA and proinflammatory cytokines. The newly recently described IRA B cells population protects against microbial sepsis, secreting IgM, IL-3, and GM-CSF. Human IRA B cells also phagocyte bacteria at least *in vitro*. Conventional CD4^+^ T cells can trans-phagocyte bacteria from infected dendritic cells (APCs) and secrete proinflammatory cytokines.

In brief, γδ T cells rapidly respond after bacteria encounter secreting cytokines regulating the immune response, similarly to cells of the innate immunity (Figure 1A). Interestingly, human γδ T cells, after activation, express molecules typically found in APCs, involved in antigen presentation, such as MHC-II and CD86 (33, 35), and it has been shown that they are able to present soluble antigens activating conventional T cells. Furthermore, some reports have also shown that human γδ T cells are able to phagocytose-opsonized beads and bacteria (36), presenting bacterial antigens on MHC class II (*in vitro*), highlighting the innate immune role of γδ T cells. However, it remains to be elucidated whether the γδ T cell-mediated antigen presentation occurs *in vivo* during the course of bacterial infections and the relevance of such antigen presentation. Moreover, it is not clear if they could elicit a memory response.

### Invariant NKT

Invariant NKT cells express an invariant TCRα chain and recognize lipid and glycolipid antigens presented by CD1d, a non-polymorphic MHC class I molecule (37). They also express several receptors, such as NK1.1 (in some mouse strains), and members of the Ly49 family that are typical of the NK cell lineage. iNKT cells are most abundant in liver, thymus, spleen, and bone marrow, but are also found in lymph nodes, peripheral blood, adipose tissue, skin, and mucosal surfaces of intestine and lungs.

It has been described that iNKT cells participate in the response to microbial pathogens in mice (38, 39) (Figure 1B). Upon activation, they produce cytokines such as IL-4 and IFN-γ. iNKTs can be activated by TCR stimulation with microbial antigens presented by CD1d (direct activation) or with endogenous antigens and/or cytokines (indirect activation) produced by APCs. The glycosphingolipid (GSL) α-galactosyl ceramide (α GalCer) was the first antigen identified recognized by mouse [Vα14 (40)] and human [Vα24 (41)] iNKT cells. Recently, it has been described a novel GSL antigen for iNKT cells, DB06-1, which induces preferentially IFN-γ *in vivo* in mice (42).

It has been reported that mouse iNKTs recognize cell wall GSL expressed by *Sphingomonas* spp., which are Gram-negative bacteria and have abundant GSLs, similar to α GalCer (43). Mice deficient of Vα14 iNKT cells present reduced spirochete clearance and are more susceptible to chronic inflammation following *Borrelia burgdorferi* infection (44). Furthermore, iNKTs recognize glycolipids expressed by *Helicobacter pilori* (45) and diacylglycerol-containing glycolipids from *Streptococcus pneumoniae* and group B *Streptococcus* (46). After infection of mice with *S. pneumoniae*, Vα14 iNKT cells produce IFN-γ, through TCR engagement (46). Recently, it has been shown that respiratory infection with *Francisella tularensis*, Gram-negative facultative intracellular bacteria that cause lethal pulmonary tularemia, activates iNKT cells which produce IFN-γ and propagates a sepsis-like proinflammatory response (47).

On the other hand, it has been demonstrated in both humans and mice that the responses by iNKT to some bacteria, such as *Salmonella typhimurium*, is due to indirect recognition of endogenous lysosomal GSL expressed by activated DCs combined with TLR activation (43, 48).

#### TLRs in iNKT Cells

It has been found that mouse iNKT cells activated by TCR resulted in increased expression of TLRs (49). In this regard, TLR4 engagement is required for production of IL-4 to further stimulate B-1 cells (50). The expression of TLR3 and 9 has been confirmed at protein level, and it has been shown that TLR signaling enhances iNKT activation. TLR stimulation of iNKT cells leads to IFN-γ, IL-4, and TNF-α production (51). On the other hand, although human iNKT cells express all TLRs, except from TLR8, they do not respond directly to TLR ligands (52).

### MAIT Cells

Mucosal-associated invariant T cells express a semi-invariant TCRα chain that recognizes small molecules, pterin analogs, and riboflavin metabolites, presented by the non-polymorphic MHC class I-related molecule, MR1 (53). MR1 is highly conserved (90% gene sequence identity between mouse and human), allowing for considerable species cross-reactivity of MAIT cells. Human MAIT cells develop effector capacity before exiting the thymus, in contrast to conventional T cells that remain naïve until antigen stimulation in the periphery (54, 55). Human MAIT cells are found principally in lungs, liver, and blood (56, 57), but they are less abundant in common laboratory mouse strains. Recently, it has been discovered that MAIT cells are more frequent in inbred CAST/EiJ mice than in C57BL/6 (58).

The role of MAIT cells in the control of bacterial infection was observed due to the absence of peripheral MAIT cells in germ-free mice and its expansion after microbial colonization (59). Both human and mouse MAIT cells can recognize bacterial (and fungi) infected cells in an MR1-dependet manner (56, 60). Human MAIT cells produce proinflammatory cytokines, e.g., IFN-γ and TNF in response to infection with *Mycobacterium smegmatis, Escherichia coli, Salmonella enterica*, or *Staphylococcus aureus* (56). However, not all microorganisms tested can activate MAIT cells. Several bacteria, such as *Enterococcus faecalis*, group A *Streptococcus*, and *L. monocytogenes*, do not stimulate human and mouse MAIT cells, neither do viruses (56, 60). Indeed, only the microorganisms that can synthetize riboflavin metabolites which bind MR1 are able to activate MAIT cells (i.e., most bacteria and some fungi) (53, 61). In agreement, MAIT cells accumulation in lungs of mice infected with *S. typhimurium* depends on microbial riboflavin synthesis (62). However, despite the fact the viruses do not produce riboflavin, viral infections (e.g., HIV) can reduce the numbers of peripheral MAIT cells (by mechanisms that are not totally understood), therefore increasing the susceptibility to opportunistic infections of bacteria and fungi (63). Nevertheless, an open question remains to be elucidated is how MAIT cells are able to recognize specific pathogens. Recently, using MR1 tetramers, it has been identified populations of MR1-restricted cells which assist to have different antigen recognition in humans (64) and mice (65).

In addition to the secretion of inflammatory cytokines, human MAIT cells achieve antibacterial immunity destroying infected cells by secretion of cytotoxic granzyme and perforin (66, 67). The role of MAIT cells during an infection *in vivo* has been demonstrated using MR1-deficient mice. These mice infected with *Klebsiella pneumoniae* develop higher bacterial burden, hypothermia, and have increased mortality in the first 4 days of infection compared with infected WT mice (68). In other infection model, using *Mycobacterium* bacillus Calmette–Guérin (BCG), MR-1 deficient mice also show higher bacterial burden in the lung compared to the WT mice (69). In both models, protection by mouse MAIT cells occurs within the first days of the infection, suggesting that they act as innate lymphocytes (Figure 1A).

Finally, it remains unresolved whether MAIT cells expressed TLRs influencing its activation or function upon stimulation.

### B Lymphocytes

It is well known that B-lymphocytes, components of the adaptive response and responsible for humoral immunity, are also APCs. They can capture soluble antigens or antigens exposed by macrophages, DCs, and follicular (FO) DCs (70). There are several subtypes of B cell lymphocytes that include B-1 (B-1a and B-1b) and conventional B-2 cells that comprise two populations designated as MZ and FO B cells.

### B-1 Cells

B-1 cells are not considered part of the adaptive immune system, as they do not develop into memory cells. B-1 cells have been identified in both human and mouse, but due to the logistical difficulties in isolating B-1 cells of humans, the vast majority of the studies have been performed in mouse models (71). B-1 resides principally in the peritoneal and pleural cavities but is in a minor fraction in lymph nodes and spleen (72). B-1 cells in the peritoneal cavity express CD11b (Mac-1) and are subdivided based on the expression of CD5.

B-1 cells play a relevant role in innate immunity by their contribution in the first line of defense against bacterial infection. B-1 cells alter their normal migration patterns (73), accumulating rapidly in the omentum, lymph nodes, and spleen, following activation by stimuli, such as IL-10, IL-5 (74), TLR agonists, such as LPSs (73, 75), or even whole bacteria, such as *S. pneumoniae* (76) or *Borrelia hermsii* (77). The exit of these cells from peritoneal cavity in response of LPS or bacteria is controlled by myeloid differentiation primary response protein 88 (MyD88), a key adaptor for TLRs signaling, that downregulates the expression of integrins and CD9, thereby promoting cell migration (73). After migration, B-1 cells differentiate and secrete rapidly large amount of IgM and/or IgA (73, 75). They are able to produce antibodies in response to T-cell-independent type 2 antigens (mainly repetitive structures from encapsulated bacteria) along with MZ B cells (76).

Regarding the phagocytic capacity of B-1 cells after bacteria encounter, there are several reports showing that B-1 cells are able to phagocyte *S. aureus, E. coli*, and polystyrene fluorescent microspheres (78, 79). Upon phagocytosis, B-1 cells kill internalized bacteria *via* phagolysosomes and present bacterial antigens in MHC-II molecules (78, 79). *Salmonella* spp. has been shown to be degraded through both proteasomal and lysosomal processing, resulting in MHC-I antigen presentation (80). On the other hand, not all bacteria are killed; some *Salmonella* can survive within B cells, using therefore B cells as Trojan horses to disseminate through the infected host (81), similarly to what has been observed in myeloid cells (82). It has been also described that B-1 cells undergo differentiation to acquire a mononuclear phagocyte phenotype *in vitro* (B-1CDP), and they are able to phagocytose *Coxiella burnetii* and kill them more effectively than peritoneal macrophages and bone marrow-derived macrophages (BMMf) (83). Moreover, it has been demonstrated that B-1 cell differentiation into phagocytes occurs also *in vivo* (84). These results revealed that mammalian B-1 cells have phagocytic and microbicidal abilities to strengthen the innate nature of these cells (Figure 1B). In agreement with the innate behavior of B1 cells, a recent report shows that during aging, mouse B1 cells express high levels of the costimulatory molecule CD86 and become potent activators of CD8^+^ T cells, a role deserved for specialized populations of APCs (85).

### Conventional B-2 Cells

The current consensus is that B-1 cells are phagocytic, whereas the phagocytic abilities of mouse conventional B cells and the mechanisms for bacterial uptake are less clear (79). BCR-blocking antibodies do not alter the internalization of bacteria, indicating that BCR is not involved in bacteria entry. In contrast to these data, it has been demonstrated that mouse liver B cells (both B-1 and B-2 cells) actively phagocytose and kill bacteria, such as *E. coli*, in a complement-dependent manner (86). Accordingly, it has been shown that splenic mouse B cells can internalize opsonized *Brucella abortus* (87), but *B. abortus* can survive inside B cells. In addition, it has also been reported that *S. typhimurium* are able to infect and survive within both mouse splenic B-1 and B-2 cell subpopulations. *Salmonella* infection stimulates expression of PD-L1 on mouse B cells, suggesting that PD-1/PD-L1 pathway may be involved in turning off the cytotoxic effector response during persistent infection (80). Furthermore, it has been reported that human primary B cells are able to internalize *S. typhimurium* (88). This process is BCR mediated and leads to efficient antigen loading into MHC-II, inducing CD4^+^ T cell help to boost *Salmonella*-specific antibody production. *Salmonella*-specific B cells that phagocytose *Salmonella* upon BCR ligation reactivate human memory CD8^+^ T cells *via* cross-presentation (89). Additionally, it has been demonstrated that both human peripheral blood and mouse splenic B-lymphocytes serve as a niche for intracellular *Salmonella* promoting systemic spreading of infection (81).

Conventional B-2 cells are divided into two populations designated as MZ and FO B cell. MZ B cells are a special population of mostly non-recirculating B cells enriched primarily in the MZ of the spleen. They are one of the first cells that take contact with blood-borne pathogens, supporting the first line of host defense. Pathogens trapped in the MZ activate MZ B cells, which maturate to plasma cells secreting IgM or to APCs. It has been reported that MZ B cells capture, process, and present antigens to T cells more efficiently than FO B cells both *in vitro* (90) and *in vivo* (91). MZ B cells appear, therefore, as excellent APCs, which, together with lymphoid DCs, play essential roles in the initial steps of *in vivo* T-cell activation. Consequently, they participate in T-cell-dependent (TD) immune response through the capture and import of blood-borne antigens to FO areas of the spleen (91).

Mice depleted of MZ B cells and infected with *B. burgdorferi* show elevated pathogen burden and reduced levels of *B. burgdorferi*-specific IgG and IgM, correlated with diminished splenic CD4^+^ T-cell responses (92). Similarly, these mice show an increased susceptibility to *S. aureus* (93). The clearance of the bacteria *L. monocytogenes* depends on the interactions between marginal zone macrophages (MZ M) and MZ B cells (94). MZ M bind pathogens and capture antigens through various PRRs, including scavenger receptors and C-type lectin receptors (CLRs) (95), and then, they expose native antigens and establish direct cell–cell contact for the activation of MZ B cells (96) that are required for potent responses (94).

#### TLRs in B Cells

B cells can interact with bacteria *via* BCRs or TLRs. The expression and functionality of TLRs in B cells has been well characterized during last years. Both mouse and human B cells express a variety of TLRs (TLR1–10) (97, 98), but mouse TLR10 is not functional.

Bacterial proteins can regulate the expression of TLR in mouse B cells, such as *Shigella dysenteriae* porin, which increases the levels of TLR2, -4, and Myd88 on peritoneal B-1 cells (99). Stimulation of TLR in B cells can modify many effectors functions, and the effects depend on the development phase of B cell. TLR4 and 9 engagements at the immature and transitional B cell stage promote proliferation and survival (100). The proliferation of peritoneal B-1 cells in response to TLR stimulation is lower than splenic B-2 cells (101). On the other hand, TLR stimulation of mature B cells promotes proinflammatory cytokines production (102, 103); MZ B cells produce IL-6 and IL-10, FO B cells secrete IFN-γ and IL-6 (98), and peritoneal B-1 cells produce high levels of IL-10, limiting the clearance of *B. hermsii* infection (101). Moreover, many surface proteins are expressed in response to TLR signaling in B cells such as the receptors for B cell-activating factor belonging to the TNF family (BAFF), an important B cell survival factor in the periphery, and APRIL (97, 104).

Toll-like receptor signaling in B cells can also result in differentiation into plasma cells or influence class switching and affinity maturation (105). TLR agonists stimulate the proliferation of mouse MZ B cells and their phenotypic maturation process, increasing MHC-II, CD40, and CD86 molecules. Depending on TLR agonist, they also secrete a distinct cytokine profile (106). TLR agonists also activate MZ B cells *in vivo* and promote the migration from the MZ, accelerating the Ag-specific IgM response (107). It is shown that p110δ activity mediates TLR-induced proliferation and antibody responses by MZ B cells (108). Recently, it has been described that TLR4 stimulation can promote activation-induced cell death (AICD) in MZ B cells, increasing FasL and Fas expression, regulating T-cell-independent B cell responses (109).

In humans, TLR ligands can promote the differentiation of transitional B cells into MZ-like B cells, and patients with defective TLR signaling have reduced numbers of MZ B cells (110).

Therefore, TLR signaling in B cells induces functional responses including cytokine, immunoglobulin production, antigen presentation, proliferation, and modulation of several surface receptors. These responses depend on the B cell development stage.

### Innate Response Activator Cells

Innate response activator (IRA) B cells have recently been described as a B cell population that protects against microbial sepsis in mice. They are accumulated in the spleen in a mouse model of sepsis and in response to *E. coli* infection, indicating that IRA B cell expansion is an overall characteristic of the body’s reaction to bacteria (111). IRA B cells are different phenotypically and functionally from other B cell populations. They contain large amounts of intracellular IgM and spontaneously secrete IgM, but not IgA or IgG1. In addition, they are able to secrete granulocyte macrophage colony-stimulating factor (GM-CSF) and IL-3 (Figure 1B). IRA B cells derived from B-1 cell precursors are activated by TLR stimuli, and they protect against septic shock by controlling neutrophil-dependent bacterial clearance (111).

B-1a cells migrate to the lung in response to microbial airway infection, producing IgM. This process is depended on IRA B cells, which controls IgM production *via* autocrine GM-CSF signaling, conferring a first-line defense against bacteria in the lungs (112).

Recently, it has been described IRA B cells in humans. They reside in tonsils, within FO areas, which are the first route of defense from infection of the upper respiratory tract and are able to phagocyte bacteria, such as *S. aureus*, at least *in vitro* (113).

Therefore, IRA B cells seem to play important roles in bacterial clearance, but further work is required to clarify its function *in vivo*, and it remains to be studied whether IRA B cells directly interact with infecting bacteria and the nature of such interactions.

### Innate Lymphoid Cells

Innate lymphoid cells are a recently identified member of the lymphoid lineage, which are enriched at epithelial barriers, such as skin, intestine, and lung, where contacts with microorganisms normally occur (114, 115). These innate lymphocytes mediate immune responses against infections and regulate homeostasis and inflammation (116, 117). They neither express TCRs or BCRs nor respond in an antigen-specific manner.

Innate lymphoid cells are divided into three subsets: group 1 ILCs (ILC1s and NK cells), group 2 ILCs (ILC2s), and group 3 ILCs (ILC3s and LTi cells). This nomenclature was unified to classify these emerging cell populations, which had been called by different terms including NK-22 cells, LTi-like cells, natural helper cells, nuocytes, and innate helper cells (118). ILCs are crucial in the protective immunity against bacteria (ILC1s and ILC3s) (119–121), intracellular parasites (ILC1s) (122), fungi (ILC3) (123), and parasitic worms (ILC2s) (124, 125).

#### Group 1 ILCs

Group 1 ILCs consisted of ILC1 and NK cells that produce IFN-γ and TNF-α after stimuli (when stimulated by IL-12, IL-15, or IL-18) (126) and had the T-box transcription factor (T-bet) as a key transcription factor (122, 127, 128). NKs were first described as innate lymphocytes with cytotoxic activity (129) that kills target cells. However, ILC1s are barely cytotoxic and seems to emerge from ILC3s (130) and are accumulated in inflamed mucosa tissue (131).

ILC1 populations have an important role in promoting defense against intracellular pathogens (Figure 1A). They secrete IFN-γ and TNF-α in mice infected with oral pathogen *Toxoplasma gondii*, recruiting myeloid cells that cease infection (122).

However, recently, it has been demonstrated that ILC1s also are important in promoting immunity to extracellular bacteria such as *Clostridium difficile* (119).

The deficiency of IFN-γ or T-bet-expressing ILC1s in Rag1^−/−^ mice increases susceptibility to *C. difficile* (119). Furthermore, it has been shown that Nfil3, an important transcription factor for the development of NKs and ILC1s, plays a role in the intestinal innate immune defense against acute bacterial infection with *Citrobacter rodentium* and *C. difficile* (132). Nfil3 deficiency results in more susceptibility to both intestinal pathogens but also corresponds to severely reduction of ILC3s and ILC2s, revealing a general requirement for this transcription factor in the development of all ILC lineages (132).

#### Group 2 ILCs

Group 2 ILCs, also referred as natural helper cells, noucytes, or innate helper 2 cells, are innate lymphocytes that produce IL-5 and IL-13 when stimulated with IL-25, IL-33, or thymic stromal lymphopoietin (TSLP) (133, 134). They were discovered after administration of IL-25 intranasally in Rag2^−/−^ mice, which lack conventional B and T cells (135, 136). ILC2s have been identified in fat tissue, spleen, nasal tissue, lung, intestine, and skin (137). ILC2 populations protect against helminth such as *N. brasiliensis* secreting IL-13 after infection (124, 133). IL-13 is necessary for the elimination of the parasite from the gastrointestinal tract, and transferring ILC2s into IL-13-deficient mice shows that IL-13 production by ILC2s is sufficient to resolve helminth infection (125). Moreover, it has been reported that ILC2s can promote IL-13-mediated immunity to other parasites in mice (138).

#### Group 3 ILC: ILC3s

Three groups characterized ILC3s in intestine as ILCs almost simultaneously (120, 139, 140). ILC3 populations secrete IL-17A, IL-22, TNF-α, and GM-CSF when activated (140–142). The transcription factor RORγt is an important regulator of this population (140). ILC3s are referred as NCR22 cells, NKp46^+^ ILCs, ILC22s, and NKR-LTi cells in the literature. This family is described in mucosal tissues, particularly in the intestinal tract, where they mediate the balance between the immune system and the symbiotic microbiota (120). It has been recently shown that mouse ILC3 cells form a functional unit together with glial cells that sense the gut environment in a MyD88-dependent manner and control the immune response *via* IL-22 secretion (143) (Figure 1A). ILC3 populations rapidly respond to infection of mice either extracellular bacteria (120, 121, 144) or fungi (123). ILC3s produce IL-22 after *C. rodentium* challenge in mice (140, 145), which is essential for host protection (146). IL-22 stimulates intestinal epithelial cells (IECs) to produce antimicrobial peptides and mucus, limiting the replication, dissemination, and tissue damage induced by pathogenic bacteria (116).

Similarly, ILC3s located in the oral mucosa produce IL-17 and IL-22 promoting immunity in mice against the fungal pathogen *Candida albicans* (123, 147). IL-17 acting alone or synergistically with IL-22 induces the recruitment of neutrophils to the site of infection. Moreover, it is shown that ILC3s also regulate neutrophils in neonatal mice, important for resistance to sepsis with Gram-negative opportunistic bacteria (148).

The production of IFN-γ by T-bet-expressing ILC3 contributes to the protection of the epithelial barrier during against *S. typhimurium* infection in mice (149). On other hand, it has been described that the expression of IL-17 and IFN-γ from ILC3s has been involved to drive inflammation in *Helicobacter hepaticus*-induced colitis (150), a mouse model of colitis. However, depletion of IL-22-producing ILCs localized in intestinal tissue results in peripheral dissemination of commensal bacteria, such as *Alcaligenes* species, promoting systemic inflammation (121). Consequently, these data indicate that ILCs regulate selective containment of lymphoid-resident bacteria to prevent systemic inflammation associated with chronic diseases.

ILC1s together with ILC3s mediate the recovery from *C. difficile* infection in mice (119). Previously, it has been suggested that ILC3s could play a role in the infection of these extracellular bacteria because the deficiency of the transcription factor Nfil3 resulted in a reduction of ILC3s with an increased of susceptibility to *C. difficile* infection (132). Nonetheless, it has been also demonstrated that ILC3s mediate protection against *S. pneumoniae* in respiratory tract (151).

#### Group 3 ILCs: LTi Cells

They are closely related to ILC3s, but their relationship is still controversial (114). LTi cells were first described in fetal and neonatal lymph nodes (152, 153), where they also showed that were crucial for lymphoid organogenesis. They are able to produce IL-17A and IL-22 mediating immunity to enteric pathogens (154, 155).

#### TLRs in ILCs

Mouse splenic ILC3s can produce IL-17 and IL-22 *in vivo* after contact with TLR2 ligands (154). Indeed, it has been shown that human RORγt^+^ ILCs (LTi-like ILC) express functional TLR2, and its stimulation with agonists induces IL-5, IL-13, and IL-22 expression in a nuclear factor κ B (NF-κB)-dependent manner (142). Recently, it has been reported that human ILCs isolated from duodenum biopsies express TLR2, 3, and 9, but only TLR3 agonists stimulate them to produce TNF-α and IFN-γ (156).

The expression of TLRs in ILC2s has yet to be identified. There is a report showing that TLRs stimulation of purified ILC2s does not induce IL-9 (157), but further studies must be done to verify the expression and functionality of TLRs in these cells.

Natural killer cells and NCR^+^RORγt^+^ ILCSs (ILC3s) may interact directly with bacteria through natural cytotoxicity receptors (NCRs), such as NKp44 and NKp46, which can be activated by components derived form commensal bacteria (158, 159).

### Conventional T Cells

In addition to the specialized lymphocyte populations with innate functions described above, we have recently described that conventional CD4^+^ T cells, the paradigm of the adaptive immunity, also play innate-like roles during bacterial infections, contrary to the current view of immunology (7). CD4^+^ T cells of both mouse and human origin are able to internalize different bacteria (pathogenic and non-pathogenic) such as *L. monocytogenes, S. aureus, E. coli*, and *S. enterica* from infected DCs, in a process called transinfection. Bacteria play a passive role in this process, driven by T cells (7); therefore, it would be more appropriate to term it transphagocytosis. Transphagocytic (ti) CD4^+^ T cells kill internalized bacteria in a manner reminiscent of innate immune cells and secrete proinflammatory Th-1 cytokines (IFN-γ, TNF-α, and IL-6) in a rapid innate-like response (Figure 1B). Furthermore, tiCD4^+^ T cells protect against bacterial infections *in vivo*, highly reducing the bacterial load found in liver and spleen 24 and 48 h after infections, contributing to the early innate immune response (7). This route of bacterial capture by T cells could be used for some pathogenic bacteria to spread. In this regard, it has been shown that T cells can serve as reservoir of bacteria *in vivo* (160–162). Moreover, *Shigella flexneri* manipulates the migration capacity of infected T cells in a type III secretion system-dependent manner (161–163). Transphagocytosis depends on T cell cytoskeleton, but the molecular mechanisms of how T cells can capture bacteria remain largely unknown. T cells are unable to directly capture bacteria (7); transphagocytosis requires T cell/DC intimate contact, and it is enhanced by antigen recognition by the TCR. On the other hand, T cells are unable to uptake latex beads from DCs, indicating that bacterial PAMPs are also involved in the transphagocytic process and suggest a role of T cell TLRs in this recently discovered process of bacterial uptake by CD4^+^ T cells.

#### TLRs in Conventional T Cells

The expression of almost all TLRs in CD4^+^ T cells, which would recognize bacterial PAMPs, has been identified at the mRNA level in CD4^+^ T cells (164, 165). However, it has been shown that activated mouse CD4^+^ T cells express TLR-3 and TLR-9 but not TLR-2 and TLR-4. Stimulation of TLR3 and 9 enhances survival in a NF-κB activation and is associated with Bcl-xL upregulation, without increased proliferation (166). On the contrary, it has been shown that TLR2 engagement induces Th1 activation in the absence of TCR stimulation, activating cell proliferation, cell survival, and IFN-γ production. IL-2 or IL-12 significantly enhances TLR-2-mediated IFN-γ production through the augmented activation of MAPKs (167). Furthermore, it has been described that TLR2 stimulation by porin of *S. dysenteriae* directly promotes CD4^+^ T cell survival and proliferation in mouse cells (168). Human-activated CD4^+^ T cells express TLR2 and TLR4 mRNA, but only activated cells show quantifiable surface expression of either TLR by flow cytometry (169). TLR2 activation, but not TLR4, promotes proliferation and IFN-γ, IL-2, and TNF-α production in activated CD4^+^ T cells, indicating its costimulatory nature. In memory CD4^+^ T cells, TLR2 expression is constitutive, and its activation leads to proliferation and IFN-γ production (169). On the other hand, TLR2 stimulation promotes Th17 differentiation both *in vivo* and *in vitro*, inducing proliferation and IL-17 production (30, 170). TLR9 stimulation in mouse CD4^+^ T cells induces NF-κB-dependent survival (166) and provides costimulation to T cells (171). TLR9 engagement, in combination with TCR activation, reduces irradiation-induced apoptosis in mouse CD4^+^ T cells and increases the rate of DNA repair (172). TLR9 stimulation in human effector CD4^+^ T cells promotes cell cycle entry (173). TLR3 stimulation also induces NF-κB, MAPK, and the survival of CD4^+^ T cells (166). On the other hand, TLR5 engagement in combination with TCR activation results in increased proliferation and production of IL-2 in human CD4^+^ T cells (174). TLR5 and TLR7/8 act also as costimulators, upregulating proliferation and IFN-γ, IL-8, and IL-10, but not IL-4, production by human CD4^+^ T cells (175). Moreover, engagement of TLR7 in human CD4^+^ T cells prevents cell cycle entry and proinflammatory cytokines production, by increasing intracellular calcium concentrations, which leads to dephosphorylation of NFATc2 and its translocation to the cell nucleus; this activates an anergic gene expression program (176).

The role of T cell TLRs in bacterial capture and their roles in the recently described innate-like functions deserve future investigations.

## Conclusion and Future Perspectives

**Figure 2:**
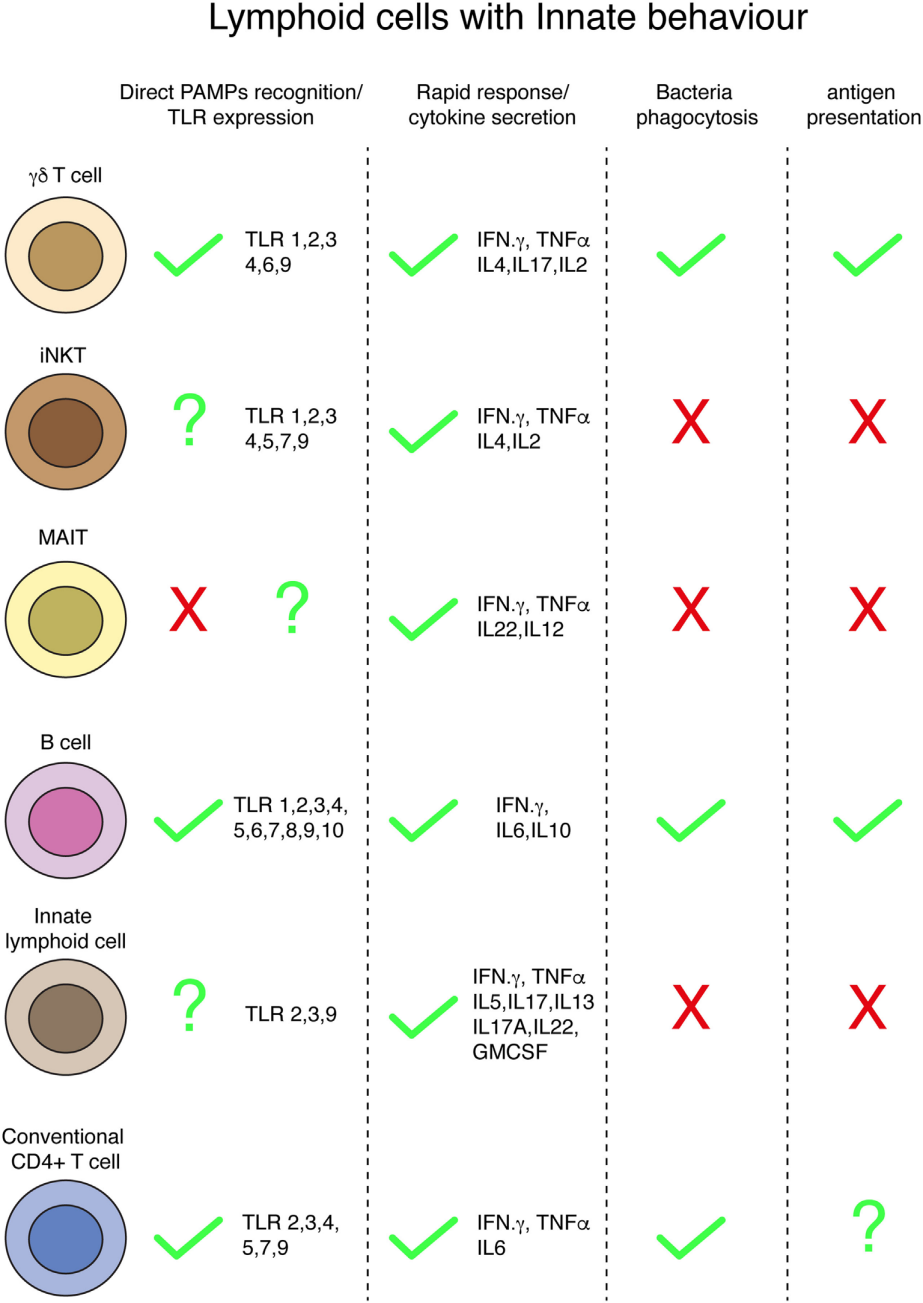
**Innate behaviors by lymphoid cells**. Summary of different innate conducts by distinct populations of lymphoid cells.

Innate, rapid responses-sensing bacteria involve complex networks of cells working in a cooperative way [e.g., ILC3-glia cells collaboration (143)]. These responses include bacteria recognition by cellular PRRs, cytokine secretion, bacteria capture and killing by phagocytosis, and antigen presentation (Figure 2). Besides classical innate immune cells, specialized populations of lymphocytes, i.e., gamma delta (γ/δ) T, iNKT, MAIT, B-1, MZ B, and IRA B cells, behave in an innate-like manner, rapidly responding upon bacteria encounter. Surprisingly, it has been demonstrated that conventional lymphocytes (both B and T cells) can internalize bacteria in an innate-like manner. CD4^+^ T cells can capture and kill bacteria by transphagocytosis from infected DCs. A similar way of bacteria capture from one infected cell to another has been also recently described for macrophages (177), and it is known from long as a mechanism from viral spread (i.e., HIV and hepatitis C virus) (178). The precise role of the CD4^+^ T cell-dependent bacterial clearance during infections *in vivo* remains to be determined, as the number of bacteria directly cleared by transphagocytosis seems to be low, suggesting other mechanisms for the reduction of bacterial load (i.e., cytokine release or antigen presentation). In agreement with this hypothesis, transphagocytic T cells secrete large amounts of proinflammatory cytokines, mounting a potent Th-1 response.

One of the hallmarks of the innate immunity is the antigen-presentation capacity of phagocytes; it has been proposed that gamma delta T cells are able to present antigen from degraded bacteria, and whether this occurs *in vivo*, and its role during infections, remains unknown. B1 cells (and B2) have the capacity of present antigens and this ability, in addition to play a major role during infections, has been used for years to study the molecular mechanisms of T cell activation occurring during the immunological synapse *in vitro*. Whether recently discovered transphagocytic T cells (7) are able to present antigens from engulfed and killed bacteria remains unsolved and deserve further investigations. Indeed, it has been demonstrated that human T cells can process and present soluble antigens to stimulate other T lymphocytes (179, 180).

A major issue of the lymphocyte’s innate-like responses is bacteria recognition. It is not fully clear which cellular receptors are involved in this process. TLRs are membrane-bound PRR involved in the recognition of extracellular PAMPs, initially characterized in innate immune cells. The expression of several TLRs has been found in the different subsets of lymphocytes, even in conventional T and B cells. Therefore, TLRs seem to be the best candidates for innate-like recognition of bacteria by lymphocytes. Bacterial recognition by lymphocytes during innate-like responses and the role that TLRs would play deserve future research. Due to the similarities in TLRs activation between bacterial PAMPs and danger signals found in malignant cells, the study of lymphocyte activation by bacteria could improve the immunotherapies against cancer.

## Author Contributions

Both EV and AC-A contribute equally to this work. Both are also co-corresponding authors.

## Conflict of Interest Statement

The authors declare that the research was conducted in the absence of any commercial or financial relationships that could be construed as a potential conflict of interest.
